# Impact of Gut Bacteria on the Infection and Transmission of Pathogenic Arboviruses by Biting Midges and Mosquitoes

**DOI:** 10.1007/s00248-020-01517-6

**Published:** 2020-05-27

**Authors:** Tim W. R. Möhlmann, Chantal B. F. Vogels, Giel P. Göertz, Gorben P. Pijlman, Cajo J. F. ter Braak, Dennis E. te Beest, Marc Hendriks, Els H. Nijhuis, Sven Warris, Barbara S. Drolet, Leo van Overbeek, Constantianus J. M. Koenraadt

**Affiliations:** 1 Laboratory of Entomology, Wageningen University & Research, P.O. Box 16, 6700 AA Wageningen, The Netherlands; 2 Department of Epidemiology of Microbial Diseases, Yale School of Public Health, 60 College Street, New Haven, CT 06510 USA; 3 Laboratory of Virology, Wageningen University & Research, P.O. Box 16, 6700 AA Wageningen, The Netherlands; 4 Biometris, Wageningen University & Research, P.O. Box 16, 6700 AA Wageningen, The Netherlands; 5 Biointeractions and Plant Health, Wageningen University & Research, P.O. Box 16, 6700 AA Wageningen, The Netherlands; 6 Bioscience, Wageningen University & Research, P.O. Box 16, 6700 AA Wageningen, The Netherlands; 7 Arthropod-Borne Animal Diseases Research Unit, USDA, Agricultural Research Service, 1515 College Ave, Manhattan, KS USA

**Keywords:** Arbovirus, Transmission, Microbiome, Biting midge, Mosquito

## Abstract

Tripartite interactions among insect vectors, midgut bacteria, and viruses may determine the ability of insects to transmit pathogenic arboviruses. Here, we investigated the impact of gut bacteria on the susceptibility of *Culicoides nubeculosus* and *Culicoides sonorensis* biting midges for Schmallenberg virus, and of *Aedes aegypti* mosquitoes for Zika and chikungunya viruses. Gut bacteria were manipulated by treating the adult insects with antibiotics. The gut bacterial communities were investigated using Illumina MiSeq sequencing of 16S rRNA, and susceptibility to arbovirus infection was tested by feeding insects with an infectious blood meal. Antibiotic treatment led to changes in gut bacteria for all insects. Interestingly, the gut bacterial composition of untreated *Ae. aegypti* and *C. nubeculosus* showed *Asaia* as the dominant genus, which was drastically reduced after antibiotic treatment. Furthermore, antibiotic treatment resulted in relatively more *Delftia* bacteria in both biting midge species, but not in mosquitoes. Antibiotic treatment and subsequent changes in gut bacterial communities were associated with a significant, 1.8-fold increased infection rate of *C. nubeculosus* with Schmallenberg virus, but not for *C. sonorensis*. We did not find any changes in infection rates for *Ae. aegypti* mosquitoes with Zika or chikungunya virus. We conclude that resident gut bacteria may dampen arbovirus transmission in biting midges, but not so in mosquitoes. Use of antimicrobial compounds at livestock farms might therefore have an unexpected contradictory effect on the health of animals, by increasing the transmission of viral pathogens by biting midges.

**Electronic supplementary material:**

The online version of this article (10.1007/s00248-020-01517-6) contains supplementary material, which is available to authorized users.

## Introduction

Symbiotic microorganisms play a key role in the physiology of their insect hosts [1, 2]. For example, microorganisms that reside in the insect gut provide extra nutrients to insects with a poor diet, such as aphids and termites [3, 4]. Furthermore, gut bacteria are important in insect development and fitness. Developmental time was delayed and egg production was reduced in mosquitoes reared free of living bacteria [5, 6]. Of particular interest is the tripartite interaction among the insect vector, their midgut bacteria, and the pathogens that these vectors may transmit [7, 8]. Midgut microbiota can provide direct protection against pathogens that enter the insect body as was shown for Triatomine bugs and malaria mosquitoes [9, 10]. For several arboviruses transmitted by *Aedes aegypti*, there is evidence for both positive and negative effects [11–14]. The beneficial bacteria have already been used in the control of arthropod-borne pathogens. Genetically modified bacterial symbionts such as *Asaia*, *Pantoea agglomerans*, *Rhodococcus rhodnii*, and *Serratia* have been used to combat pathogen transmission by vectors [12, 15–20]. In addition, the endosymbiotic bacterium *Wolbachia* is a well-studied example of how a microbe can disrupt the transmission of arboviruses by mosquitoes [21–24]. However, a study conducted on the interaction of *Wolbachia* and West Nile Virus in *Culex* mosquitoes showed increased virus titers in the presence of *Wolbachia* [25]. This indicates that the impact of bacteria on virus transmission is context-dependent.

Although the effects of *Wolbachia* and several genetically modified bacteria on arbovirus transmission have been extensively studied [11, 21, 24], thus far, only few studies have investigated the role of symbiotic midgut bacteria on pathogen transmission [19]. Pathogens are ingested together with a blood meal and have to overcome the midgut barrier before they can infect the insect body. It is hypothesized that midgut bacteria have an effect on pathogen infection either mechanically or via activation of the vector’s immune system. While the interaction of mosquito midgut bacteria with malaria parasites has been studied in more detail [14, 16, 26–29], relatively few studies have investigated the role of microbiota in transmission of arboviruses. Previous reports have shown an increased replication of arboviruses after elimination of the midgut bacteria [12–14, 30–32]. At the individual level, elimination of the mosquito midgut bacteria seems to reduce basal levels of antiviral immune response pathways such as the Toll pathway [30], leading to increased susceptibility to arbovirus infection. Although these studies show increased viral titers and reduced immune response pathways, they did not report on infection rate or transmission efficiency, which are important (quantitative) components of vector competence. Knowledge on the impact of the midgut bacterial community on the proportion of vectors that can transmit an arbovirus (vector competence) is currently lacking.

The aim of this study was to investigate the effect of gut bacteria on infection and transmission of arboviruses by their vector. As a model system, we selected three arboviruses belonging to different families, namely Schmallenberg virus (SBV; family Peribunyaviridae, genus *Orthobunyavirus*), Zika virus (ZIKV; family Flaviviridae, genus *Flavivirus*), and chikungunya virus (CHIKV; family Togaviridae, genus *Alphavirus*). Infection with SBV can result in symptoms ranging from short fevers and diarrhea to severe clinical manifestations, such as congenital malformation in ruminants. Infections with ZIKV and CHIKV can cause mild (fever, arthralgia) to severe disease (microcephaly) in humans. In the model systems, the arboviruses are transmitted by biting midges (SBV by *Culicoides nubeculosus* and *C. sonorensis*), or mosquitoes (ZIKV and CHIKV by *Aedes aegypti*). We sequenced the bacterial content in the gut system of both untreated and antibiotic-treated adult females of the three vector species, to identify changes in the microbial community. Subsequently, we determined infection rates and transmission efficiencies of untreated and antibiotic-treated virus-exposed females. In addition, virus titers were compared between the two treatments to investigate the effect of the gut microbial communities on the replicative fitness of the viruses.

## Methods

**Fig. 1:**
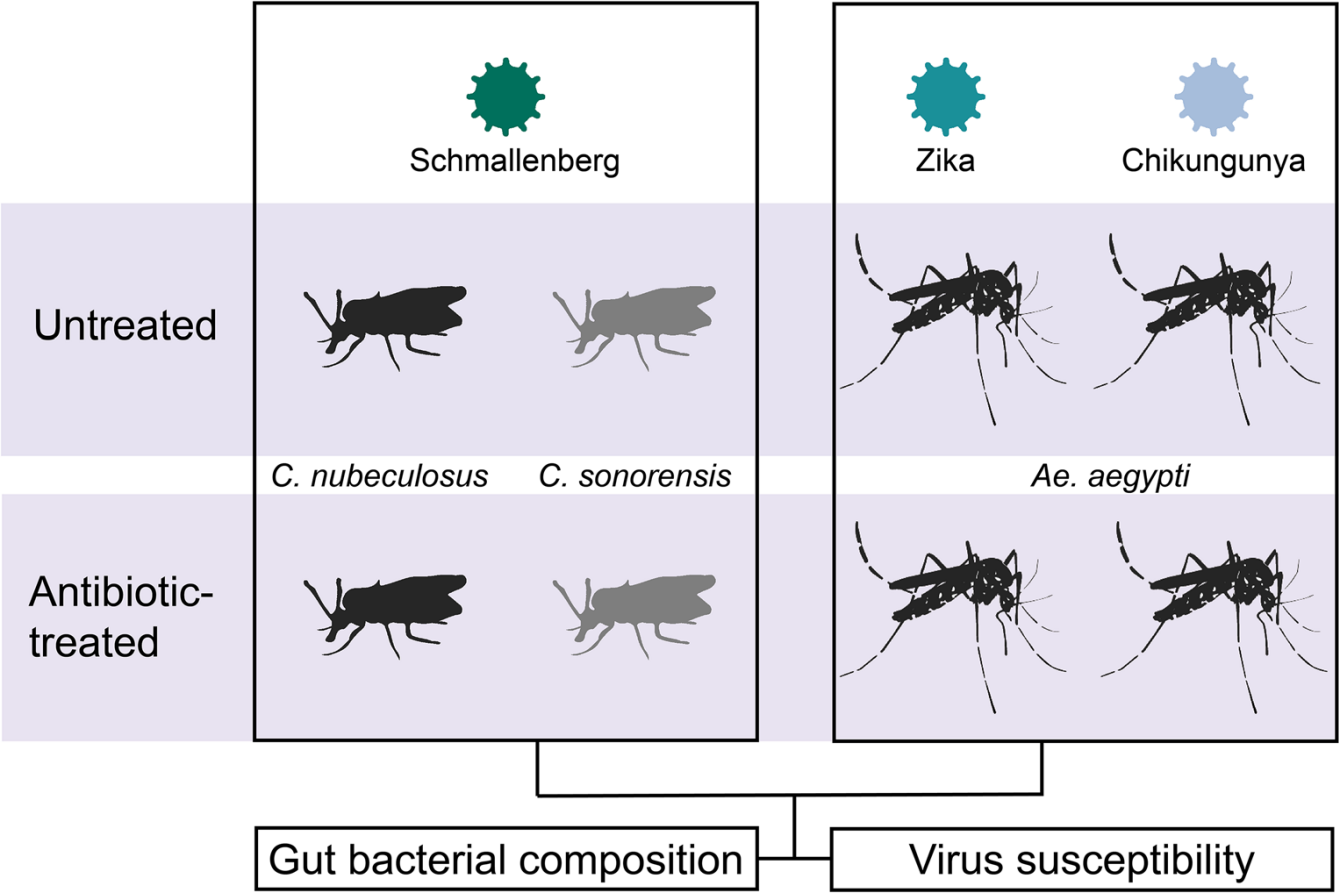
Overview of experimental design. Schmallenberg virus was used for infection of *Culicoides nubeculosus* and *C. sonorensis* biting midges, whereas Zika and chikungunya viruses were used for infection of *Aedes aegypti* mosquitoes. All three vector species were divided in an untreated and an antibiotic-treated group. The gut bacterial communities of the three vector species for the untreated and antibiotic-treated groups were identified via 16S rRNA sequencing

To investigate the role of insect midgut bacterial communities on arbovirus infection and transmission, we selected three vector species and three viruses (Fig. 1). The microbial gut communities of antibiotic-treated and untreated adult females were identified and their susceptibility to the respective viruses was tested. Two biting midge species (*C. nubeculosus* and *C. sonorensis*) were exposed to SBV. One mosquito species (*Ae. aegypti*) was exposed to ZIKV or CHIKV. Adult female insects of each species were divided into untreated (control) and antibiotic-treated groups.

### Insect Vectors

*Culicoides nubeculosus* were provided by The Pirbright Institute, Pirbright Laboratories, UK [33], and were maintained at 23 ± 1 °C with 16:8 light:dark cycle and 60% relative humidity. *Culicoides sonorensis* were provided by the Arthropod-Borne Animal Diseases Research Unit, USDA-ARS and were maintained at 25 °C with 16:8 light:dark cycle and 70% relative humidity. Similar rearing protocols were used for both biting midge species [34]. Briefly, eggs were transferred to trays with filter wool pasted to the bottom (Europet Bernina International, Gemert-Bakel, The Netherlands), filled with tap water and two drops of Liquifry No.1 (Interpet, Dorking, UK). Larvae were fed with a 1:1:1 mixture of bovine liver powder (MP biomedicals, Irvine, CA, USA), ground rabbit food (Pets Place, Ede, The Netherlands), and ground koi food (Tetra, Melle, Germany). *Culicoides nubeculosus* larvae were additionally fed with nutrient broth No. 1 (Oxoid, Hampshire, UK). Pupae were transferred to moist emergence cups that were placed in plastic buckets (diameter 12.2 cm, height 12.2 cm; Jokey, Wipperfürth, Germany) and closed with netting on the top through which the biting midges could feed. Emerged adults were provided with 6% glucose solution ad libitum. Antibiotic free, bovine blood (Carus, Wageningen, The Netherlands) was provided through a Parafilm M membrane using the Hemotek PS5 feeding system (Discovery Workshops, Lancashire, UK) for egg production.

*Aedes aegypti* from the Rockefeller strain (Bayer AG, Monheim, Germany) were used in all mosquito experiments. The mosquito colony was maintained as described previously [35]. In short, mosquitoes were kept at 27 ± 1 °C with 12:12 light:dark cycle and 70% relative humidity. Adult mosquitoes were maintained on 6% glucose solution ad libitum. Antibiotic free, human blood (Sanquin Blood Supply Foundation, Nijmegen, The Netherlands) was provided through a Parafilm M membrane using the Hemotek PS5 feeding system for egg production. Drought-conditioned eggs were transferred to transparent square larval holding trays (19 × 19 × 20 cm; Jokey), filled for approximately one-third with tap water and three drops of Liquifry No. 1. Hatched larvae were fed with TetraMin Baby fish food (Tetra). Larval trays were closed with fine-meshed netting to allow adult mosquitoes to emerge inside larval trays. Twice a week, adults were aspirated from larval trays and collected in Bugdorm-1 insect rearing cages (30 cm × 30 cm × 30 cm; BugDorm, Taiwan, China).

### Antibiotic Treatment

Approximately 100–200 *C. nubeculosus* and *C. sonorensis* pupae were collected during three consecutive days and placed in a Petri dish containing moistened cotton wool and filter paper in separate buckets (diameter 12.2 cm, height 12.2 cm; Jokey). For a period of 6 days, they were allowed to emerge and had direct access to 6% glucose solution (untreated group), or 6% glucose solution containing a combination of 10 μg/ml penicillin and 10 μg/ml streptomycin (Duchefa Biochemie B.V., Haarlem, The Netherlands) (antibiotic-treated group) [36]. Penicillin was chosen because it is a broad-spectrum antibiotic against gram-positive bacteria, and streptomycin was chosen because it is a broad-spectrum antibiotic against gram-negative bacteria. Biting midges in the antibiotic-treated group were allowed to feed on a glucose solution with antibiotics for 3 to 6 days before being transferred to the Biological Safety Level 3 (BSL3) facility at Wageningen University & Research, where arbovirus infections were performed. Antibiotic treatment was continued throughout the duration of the experiments.

*Aedes aegypti* adults were collected from larval trays and divided into two groups of approximately 100–200 mosquitoes in Bugdorm-1 cages. One cage was maintained on 6% glucose solution (untreated group), whereas the other cage was maintained on 6% glucose solution with 20 U/ml penicillin (Sigma-Aldrich, Saint Louis, MO, USA) and 20 μg/ml streptomycin (PenStrep) (antibiotic-treated group; Sigma-Aldrich) for 4 days [32]. Females were then transferred to plastic buckets (diameter 12.2 cm, height 12.2 cm; Jokey) and transported to the BSL3 facility for arbovirus infection studies.

### Taxonomical Identification of Gut Bacterial Populations

#### Sample Preparation

To gain insight in the effect of the antibiotic treatment on gut bacterial community composition, biting midges and mosquitoes were dissected and their gut bacterial communities were identified. Prior to dissection, biting midges were anesthetized by freezing for 15 to 30 min at − 20 °C. To remove external bacterial contamination, each biting midge was dipped in 70% ethanol for 10 s, in 5% sodium hypochlorite solution for 60 s, and finally rinsed in 70% ethanol for 30 s [37–39]. After cleaning, pools were made under aseptic conditions from dissected abdomens from five untreated or five antibiotic-treated females in a 2-ml screw cap micro-tube (Sarstedt) with a 4-mm borosilicate glass bead (Sigma-Aldrich). In total, 18 replicate pools were prepared for untreated and antibiotic-treated *C. nubeculosus*, and 10 replicate pools for untreated and antibiotic-treated *C. sonorensis*, resulting in a total of 56 pools.

Similar to the biting midges, mosquito midgut bacteria were investigated. Mosquitoes were treated in a similar manner as described above. Midguts were dissected and pooled from five untreated and five antibiotic-treated females, under aseptic conditions. Selected mosquitoes were anesthetized on ice, dipped in 70% ethanol for 10 s, and then rinsed in phosphate buffered saline (PBS) for 10 s. Midguts were dissected in a droplet of PBS using forceps, under the dissecting microscope. Five midguts per treatment were pooled in a 2-ml screw cap micro-tube with a 4-mm borosilicate glass bead. In total, 12 replicate pools were prepared for untreated and antibiotic-treated *Ae. aegypti* females, resulting in a total number of 24 pools.

#### DNA Extraction Protocol

Midgut pools were placed in Precellys Evolution tissue homogenizer (Bertin Instruments, Montigny-le-Bretonneux, France) and homogenized twice at 7800 rpm for 15 s. The Mag-Bind Tissue DNA KF 96 Kit (Omega Bio-tek, Norcross, GA, USA) was used for DNA extraction of bacterial populations as per the manufacturer’s protocol.

#### qPCR

Midgut bacterial loads were quantified for each sample by SYBR Green real-time PCR (Thermo Fisher Scientific, Waltham, USA) to estimate the relative abundance of major taxonomic groups of bacteria [40]. For each sample, 5 μl was added to a master mix of 20 μl consisting of 0.12 μl 100 μM Eub338f forward primer, 0.12 μl 100 μM Eub518r μl reverse primer, 10 μl Takara 2×, 0.4 μl ROX2, and 4.36 μl Milli-Q water. The qPCR program was run at 50 °C for 2 min, 95 °C for 10 min, then 40 cycles of 95 °C for 15 s and 50 °C for 1 min, followed by a final melting and annealing step of 95 °C for 30 s and finally 50 °C for 15 s. Then, these qPCR amplicons were run on gel and the intensity of electrophoresis was used to visually estimate if bacterial DNA load after PCR was comparable among samples. If this was not the case, this process was repeated with adjusted numbers of PCR cycles until comparable DNA load was achieved. Midge DNA extracts were then subjected in triplicate to PCR with 5 μl sample and 20 μl master mixture consisting of 1.2 μl dNTP (5 mM), 6 μl 5xQ5 reaction buffer, 0.15 μl 16S V4 515F forward primer (100 μM), 0.15 μl 16S V4 806R reverse primer (100 μM), 0.3 μl Q5 HF DNA polymerase, and 14.7 μl Milli-Q water [41]. Samples were run on Verity PCR machines (Thermo Fisher Scientific, Waltham, USA) with the following program: 98 °C for 30 s, 98 °C for 10 s, 50 °C for 30 s, 72 °C for 30 s, 72 °C for 2 min, and 4 °C until the program was stopped. The number of cycles varied per sample but all were between 16 and 29 cycles. Obtained amplicons of the three PCR replicates per sample were pooled and stored at − 20 °C before further processing.

### Sequencing and Preparation of Data

Samples were sequenced on an Illumina MiSeq platform (Next Generation Sequencing Facilities, Wageningen University & Research, Wageningen, The Netherlands). Resulting reads were analyzed with QIIME2 (version 2018.8; https://qiime2.org; [42, 43]). All forward and reverse reads were demultiplexed and linked to sample IDs. Sequence run specific quality control, merging of forward and reverse reads, removal of 16S V4 primer sequences and of chimeric sequences was performed with the DADA2 package as QIIME2 plugin [44]. DADA2 grouped unique sequences equivalent to operational taxonomic unit (OTU) clustering at 100% similarity, resulting in an abundance table (feature table) of the amplicon sequence variants (ASVs) and a file with the unique sequences (rep-seqs). Advantages of the new ASV approach compared with OTU clustering at 97% similarity have been discussed previously [45]. At first, sequences were aligned with MAFFT plugin [46] and highly variable positions in alignment were masked [47] to reduce noise in the phylogenetic tree. FastTree plugin [48] was used to create an unrooted tree of the unique sequences. The tree was rooted at midpoint of the longest tip-to-tip distance.

Taxonomy was assigned with confidence threshold 0.8 to the unique sequences with Naive Bayes classifier pre-trained on the Silva database release “132 16S V4 region,” with QIIME2 classifier plugin (https://docs.qiime2.org/; [49–51]. The ASV abundance table was additionally filtered before further analyses. All sequences were removed that were not classified (unassigned at Kingdom taxa level) or classified as Eukaryotes, plant mitochondria, or chloroplasts, as well as all ASVs without any phylum classification. Very low abundant ASVs with a total count below 10 were also removed as an additional noise reduction before further analyses. For analyses performed in R, the QIIME2 data was extracted into abundance or feature tables and converted from BIOM HDF5 to JSON format [52].

### Negative Control Samples

Negative control samples (*N* = 14) were included that followed the complete protocol from DNA extraction to sequencing. These samples contained no insect material but did generate bacterial sequences. Such contaminants can originate from reagents used in the DNA extraction, PCR, or next-generation sequencing library preparation, as well as from human skin, oral, or respiratory microbiota [53, 54]. The 14 samples contained 907 ASVs with a count of 204,153. After filtering of low abundant ASVs, a total of 81 ASVs with a count of 176,725 remained. To identify true contaminants, an occurrence threshold of 20% was used which means that an ASV was present in at least 3 out of the 14 negative control samples. In addition, the selected contaminants together had to contribute 99% to the total fraction counts. A total of 51 ASVs with a count of 140,573 were recognized as true contaminations and filtered from the complete dataset before further analyses. Identified contaminants consisted of several common skin bacteria such as *Corynebacteria*, *Propionibacteria*, *Staphylococci*, and *Micrococcus* [55]. Together, these skin-associated ASVs comprised 20% (28,562/140,573) of the total count in the negative controls (Additional File S2).

### Viruses

SBV was obtained from Wageningen Bioveterinary Research (Lelystad, The Netherlands) as passage three (P3) bovine isolate (B-SBV). Two additional passages, P4 and P5, were grown on *Aedes albopictus* C6/36 cells (ATCC, Manassas, USA, CRL-1660) in Leibovitz-15 (L-15) growth medium (Gibco, Carlsbad, CA, USA) supplemented with 10% fetal bovine serum (FBS), 2% tryptose phosphate broth (Gibco), and 1% nonessential amino acids (Gibco), at 27 °C. Virus-containing supernatants were harvested at 5 days post-inoculation and stored in aliquots at − 80 °C. The P4 stock titer was determined by endpoint dilution assays (EPDA) on African green monkey kidney Vero E6 cells (ATCC CRL-1586). Virus titers were determined using the Reed and Muench algorithm [56].

ZIKV Suriname strain P4 stock, as described previously by Göertz et al. [35], was used to grow a P5 stock on Vero cells and was used in all mosquito infection experiments. Vero cells were cultured in Dulbecco’s modified Eagle medium (HEPES-DMEM; Gibco) supplemented with 10% FBS, at 37 °C, and 5% CO_2_. A T75 flask (Greiner Bio-One, Kremsmünster, Austria) pre-seeded with Vero cells was inoculated with ZIKV P4, and incubated for 3 days. Supernatant was harvested and stored in aliquots at − 80 °C. The P5 stock titer was determined by EPDA, as described above, on Vero cells.

Chikungunya strain 37997 was produced as previously described [35]. A T75 flask pre-seeded with C6/36 cells was inoculated with CHIKV P1 and incubated for 3 days at 28 °C. Supernatant was harvested and stored in aliquots at − 80 °C. The P2 stock titer was determined by EPDA, as described above, on Vero cells.

### Virus Infections

Untreated and antibiotic-treated female biting midges were allowed to feed on an infectious blood meal containing SBV, whereas female mosquitoes were allowed to feed on an infectious blood meal containing either ZIKV or CHIKV. For each virus, a 1:1 dilution was prepared by adding an equal amount of bovine blood to SBV stock (average titer in blood meal, 2.5 × 10^6^), or human blood to either ZIKV stock (titer in blood meal, 4.0 × 10^4^) or CHIKV stock (titer in blood meal, 2.5 × 10^8^). These virus titers were deliberately selected based on pilot experiments to obtain intermediate infection rates to facilitate observations of both negative and/or positive effects of the midgut bacteria on virus infection rates. Bovine blood was verified for absence of SBV neutralizing antibodies before the experiment started. The infectious blood meal was provided through a Parafilm M membrane using the Hemotek PS5 feeding system, at 24 ± 1 °C and 70% relative humidity. Biting midges were fed in the dark, whereas mosquitoes were fed under light conditions. After 1 h, biting midges and mosquitoes were anesthetized with 100% CO_2_, placed on a CO_2_-pad (Genesee Scientific, San Diego, USA), and fully engorged females were selected and placed back in the holding bucket. Biting midges were maintained at 25 °C for 10 days and provided with 6% glucose solution ad libitum (untreated). Biting midges in the antibiotic-treated group were continuously fed on the glucose solution with PenStrep (antibiotic-treated). Engorged female mosquitoes in both treatments were maintained at 28 °C for 10 days and were provided with 6% glucose solution ad libitum.

### Infection and Transmission

Ten days post-feeding, biting midges were anesthetized with 100% CO_2_ and maintained on a CO_2_-pad. Females were individually transferred to a 1.5-ml Safe-Seal micro-tube (Sarstedt, Nümbrecht, Germany) containing 0.5 mm zirconium beads (Next Advance, Averill Park, NY, USA) and stored at − 80 °C until further processing. The whole procedure was replicated three times, which resulted in a total number of 196 untreated (*N*_1_ = 79, *N*_2_ = 49, *N*_3_ = 68) and 275 antibiotic-treated (*N*_1_ = 114, *N*_2_ = 92, *N*_3_ = 69) C*. nubeculosus*, and 44 untreated (*N*_1_ = 20, *N*_2_ = 10, *N*_3_ = 14) and 47 antibiotic-treated (*N*_1_ = 19, *N*_2_ = 5, *N*_3_ = 23) *C. sonorensis*.

Ten days post-feeding, mosquitoes were anesthetized with 100% CO_2_ and maintained on a CO_2_-pad to remove their legs and wings with forceps. Mosquito saliva was then collected by inserting the proboscis into a 200-μl yellow pipet tip (Greiner Bio-One) containing 5 μl of a 1:1 solution of 50% glucose solution and FBS. After at least 45 min, the mosquito body (head, thorax, and abdomen) was transferred to a 1.5-ml Safe-Seal micro-tube containing 0.5-mm zirconium beads. The saliva sample was transferred to a 1.5-ml micro-tube (Sarstedt) containing 55 μl 4-(2-hydroxyethyl)-1-piperazineethanesulfonic acid-buffered DMEM (HEPES-DMEM) supplemented with 10% FBS, penicillin (100 U/ml), streptomycin (100 μg/ml), fungizone (50 μg/ml; Invitrogen, Carlsbad, USA), and gentamycin (50 μg/ml; Gibco). All samples were stored at − 80 °C until further processing. This whole procedure was replicated four times for both ZIKV and CHIKV, with *N* = 25 mosquito body and saliva samples per replicate for each of the four treatments.

Frozen biting midge and mosquito bodies were homogenized for 2 min at maximum speed in a Bullet Blender Storm (Next advance, Averill Park, NY, USA), centrifuged briefly, and re-suspended in 100 μl of fully supplemented HEPES-DMEM. Samples were blended again for 2 min at maximum speed, and centrifuged for 2 min at 14,500 rpm in an Eppendorf minispin plus (Eppendorf, Hamburg, Germany). Mosquito saliva samples were thawed at room temperature. In total, 30 μl of each body or saliva sample was used to inoculate a monolayer of pre-seeded Vero cells in a 96-well plate. On each plate, diluted virus stock or infectious blood mixture was included as positive controls and wells to which no sample was added were included as negative controls. After 2–3 h, the inoculum was removed and replaced by 100 μl of fully supplemented HEPES-DMEM. Wells were scored for virus-induced cytopathic effect (CPE) at 3 and 6 days post-inoculation, with full CPE being observed at the latter time point. Virus titers of infected biting midge bodies and of mosquito body and saliva samples were determined by EPDA on Vero E6 cells [35]. If less than three wells in the first row showed CPE, the titer could not be calculated because the sample contained less than 1000 TCID_50_ per ml.

### Statistical Analysis

The difference in bacterial communities between untreated and antibiotic-treated insects (biting midges or mosquitoes) was tested using a permutation test (999 permutations) based on a redundancy analysis (RDA) of taxa on the treatment factor using Canoco 5.11 [57]. All seven taxonomic levels were used simultaneously in these analyses, obtained by summing the ASV counts to the taxon levels kingdom (bacteria and archaea), phylum, class, order, family, genus, and species. In the analysis, the resulting counts were divided by the library size and the resulting fractions were log-transformed after addition of 0.001, to avoid problems with zero counts. The value 0.001 was chosen as its inverse is close to the smallest library size and gives a reasonably symmetric distribution of residuals. The approach has the advantage of yielding one test of significance instead of several level-specific tests. Selection of differentially expressed taxa was based on the percentage fit due to the treatment factor (in our case antibiotic treatment).

In addition, we conducted a univariate test using the log-transformed fractions per taxon, to identify taxa that were correlated with untreated or antibiotic-treated samples. Univariate *p* values were calculated with both Welch’s two-sample *t* test (two-sided) and its permuted version. The null distribution of the permuted *t* test was calculated with 9,999 permutations with the function perm.*t*.test from the R package deducer. Given the correspondence between the *p* values of these two methods, the false discovery rate (FDR, Benjamini-Hochberg correction) was based on the *p* values of (parametric) Welch’s *t* test. The FDR was calculated across all taxa levels together and per taxon level. Alpha diversity indices were calculated for Shannon-Wiener Diversity (*H*′), the Inverse Simpson Index (D2 or N2), and the Shannon-Wiener Evenness index–based *N*1/*N*2, where *N*1 = exp(*H*′) and *N*2 = Inverse Simpson Index using the VEGAN version 2.9.2. package [58] in the statistical software package R version 3.5.0 [59].

Chi-square tests were used to test for the effect of antibiotic treatment on infection rate and transmission efficiency. For biting midges, only infection rates were determined, whereas both infection rates and transmission efficiency were determined for mosquitoes. Infection rate and transmission efficiency were calculated, respectively, by dividing the number of female vectors with virus-infected whole body (infection) or virus-infected saliva (transmission) by the total number of alive female vectors tested in the respective treatment, and multiplied by 100. Mann-Whitney *U* tests were used to test for the effect of antibiotic treatment on virus titers of body or saliva samples. All statistical analyses were done with the statistical software package R [59].

## Results

### Gut Bacterial Communities

To gain insight in the effect of the antibiotic treatment on the composition of gut bacterial communities, the identities of gut bacteria populations in adult female *C. nubeculosus*, *C. sonorensis*, and *Ae. aegypti* were determined by high-throughput 16S rRNA gene sequencing before blood-feeding. In addition, to uncover the role that specific gut bacteria may play in virus infection, bacterial species that were significantly different between untreated and antibiotic-treated females were determined by Redundancy analyses (RDA).

**Fig. 2:**
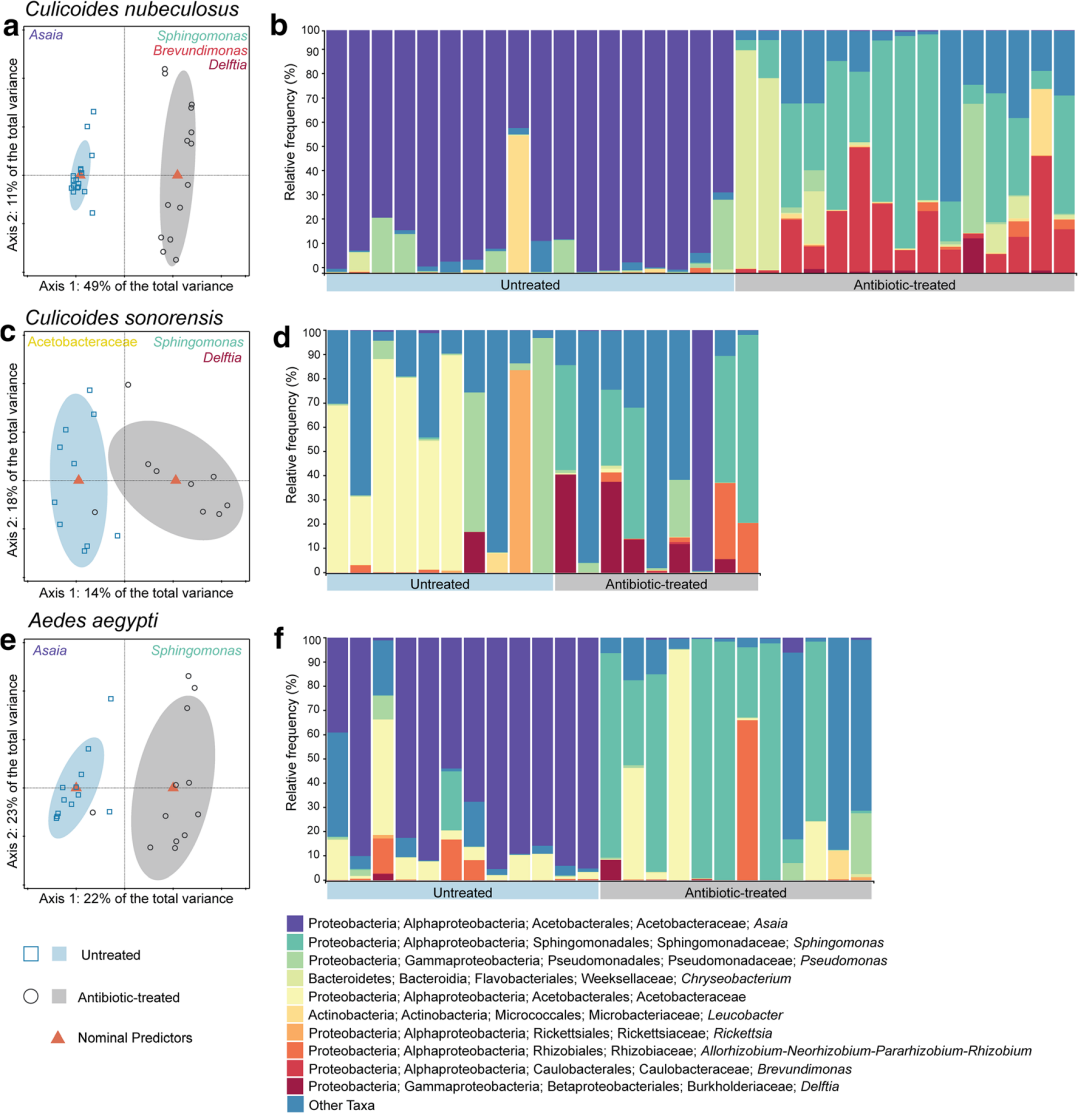
Overview of bacterial communities in untreated and antibiotic-treated biting midges and mosquitoes. RDA of logarithm of the fraction of bacteria in untreated and antibiotic-treated females of *Culicoides nubeculosus* (panel **a**; *N* = 33, *DF* = 1, *F* = 30.0, *p =* 0.001), *C. sonorensis* (panel **c**; *N* = 19, *DF* = 1, *F* = 2.7, *p =* 0.001), and *Aedes aegypti* (panel **e**; *N* = 24, *DF* = 1, *F* = 6.1, *p =* 0.001). Ellipses show 66% confidence levels (± 1 time the standard deviation). A maximum of three taxa correlated with the untreated or antibiotic-treated groups are named at the top of panels **a**, **c**, and **e** for each species. **b**, **d**, **f** Taxa-plots at genus level, on the relative frequency for each taxon, of the total number of midgut bacteria in the community composition are presented. The 10 most abundant bacterial taxa are presented for midgut bacterial communities in *C. nubeculosus*, *C. sonorensis*, and *Ae. aegypti*. Less abundant taxa were grouped as “Other taxa” to increase visualization for the taxa plots. Each bar represents the relative frequency of bacterial taxa in one pool of five abdomens

The communities of gut bacteria were significantly different between untreated and antibiotic-treated groups for all three vector species (*p* < 0.01; Fig. 2a, c, e) The first principal component (PC), reflecting the difference between the bacterial communities of untreated and antibiotic-treated mosquitoes or biting midges, could explain a large part of the total variance (Fig. 2a, c, e). There was a significant difference between the gut bacterial communities of untreated and antibiotic-treated for *C. nubeculosus* (*p =* 0.001; Fig. 2a, the first PC explained 49% of the total variation), *C. sonorensis* (*p =* 0.001; Fig. 2c, the first PC explained 14% of the total variation), and *Ae. aegypti* (*p =* 0.001; Fig. 2e, the first PC explained 22% of the total variation).

After antibiotic treatment, a clear shift in gut microbial community was observed in *C. nubeculosus* and *Ae. aegypti* (Fig. 2b, f). Untreated samples of these two species were dominated by a single ASV that had a relative frequency of 34 to 98% of the total bacterial community (Fig. 2b, f). This ASV was identified as gram-negative *Asaia* bacterium (Phylum: Proteobacteria; family: Acetobacteraceae). The gut bacterial community of *C. nubeculosus* and *Ae. aegypti* that were treated with antibiotics still contained *Asaia*, but only up to 3% of the total bacterial community. A shift in midgut bacterial species was less evident for *C. sonorensis*, which overall showed more variation in bacterial communities in both the untreated and antibiotic-treated groups (Fig. 2d). Interestingly, the diversity of bacteria was higher for all three insect species after antibiotic treatment compared with the untreated group (Table 1).

**Table 1:** Gut microbial diversity. Estimators of taxonomic diversity for gut microbiota of *Aedes aegypti*, *Culicoides nubeculosus*, and *C. sonorensis* kept on either 6% glucose solution (untreated) or 6% glucose solution with penicillin and streptomycin (antibiotic-treated). Average values (minimum–maximum) are presented for Inverse Simpson Index, Shannon-Wiener Diversity, and Shannon-Wiener Evenness

Taxonomic diversity	Mosquitoes		Biting midges			
	*Aedes aegypti*		*Culicoides nubeculosus*		*Culicoides sonorensis*	
	Untreated	Antibiotic-treated	Untreated	Antibiotic-treated	Untreated	Antibiotic-treated
No. of samples	12	12	18	15	10	9
Inverse Simpson Index	1.501 (1.009–3.063)	3.038 (1.031–11.702)	1.257 (1.011–2.070)	2.915 (1.237–5.901)	2.437(1.074–7.647)	3.135 (1.014–8.401)
Shannon-Wiener diversity	0.527 (0.039–1.720)	1.055 (0.114–3.009)	0.351 (0.046–0.837)	1.339 (0.528–2.150)	1.070 (0.202–2.771)	1.265 (0.061–3.289)
Shannon-Wiener evenness	1.236 (1.031–1.823)	1.385 (1.087–1.940)	1.158 (1.036–1.332)	1.447 (1.302–1.730)	1.522 (1.139–2.090)	1.468 (1.048–3.192)

The family Acetobacteraceae was associated with each untreated group of insects, and more specifically for both *C. nubeculosus* and *Ae. aegypti*, the genus *Asaia* within the Acetobacteraceae family. The antibiotic-treated groups for all three vector species were represented by the presence of bacteria in the *Sphingomonas* genus when compared with the untreated groups. In addition, *Delftia* bacteria were correlated with antibiotic-treated biting midges (Fig. 2).

### Infection Rates and Transmission Efficiency

**Fig. 3:**
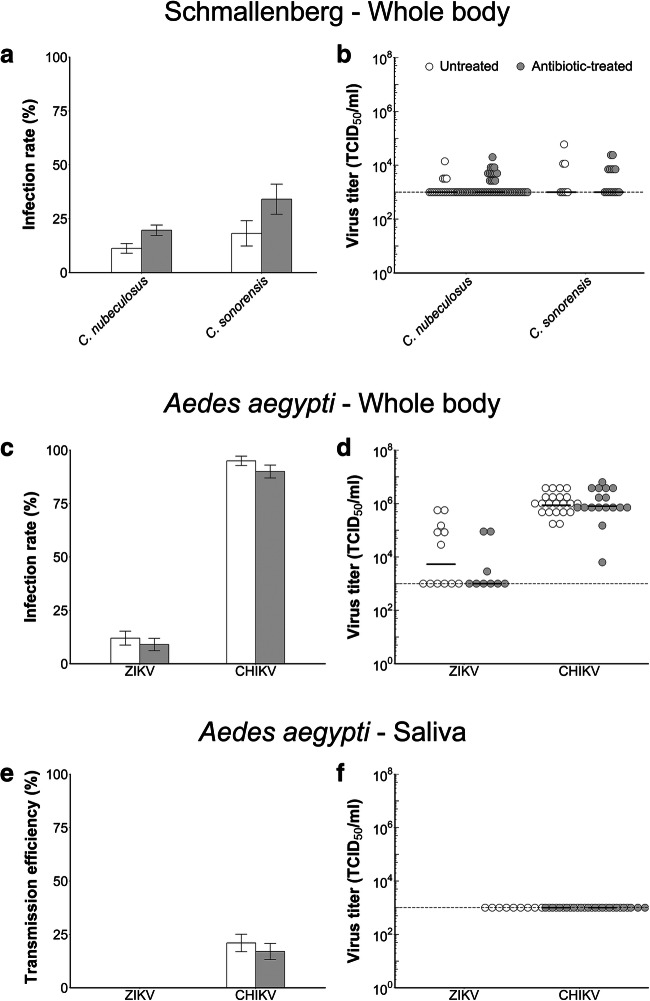
Effect of antibiotic treatment on susceptibility of biting midges and mosquitoes for arthropod-borne viruses. **a** Mean infection rates of Schmallenberg virus (SBV) in biting midges (*N*_*nubeculosus* = 196, *N*_*sonorensis* = 44; untreated: white bars) fed on glucose solution and glucose solution with antibiotics (*N*_*nubeculosus* = 275, *N*_*sonorensis* = 47; antibiotic-treated: gray bars). *Culicoides nubeculosus* and *C. sonorensis* were blood-fed 3 to 6 days after emergence and tested for virus infection after an incubation period of 10 days. Error bars indicate the SEM. **b** Average titers of SBV in infected biting midges (*C. nubeculosus* and *C. sonorensis*) for both treatments (untreated: white dots and antibiotic-treated: gray dots). Each dot represents the titer for one individual biting midge and horizontal bars indicate the median. **c** Mean infection rates of Zika virus (ZIKV) and chikungunya virus (CHIKV) in *Aedes aegypti* mosquitoes (*N* = 100 for each group) fed on glucose solution (untreated: white bars) and glucose solution with antibiotics (antibiotic-treated: gray bars). Mosquitoes were blood-fed 4 to 8 days after emergence and tested for virus infection after an incubation period of 10 days. Error bars indicate the SEM. **d** Average titer of ZIKV and CHIKV in infected mosquitoes for both treatments (untreated: white dots and antibiotic-treated: gray dots). Each dot represents the titer for one individual mosquito and horizontal bars indicate the median. **e** Percentage positive saliva samples (transmission efficiency) for untreated (white bars) and antibiotic-treated (gray bars) *Ae. aegypti* mosquitoes exposed to ZIKV or CHIKV (*N* = 100 for each group). Error bars indicate the SEM. **f** Average titer of ZIKV- and CHIKV-positive saliva samples of untreated (white dots) and antibiotic-treated (gray dots) *Ae. aegypti* mosquitoes. No positive saliva samples were found for ZIKV-infected mosquitoes. Each dot represents the titer for one individual mosquito and horizontal bars indicate the median

Vector competence was determined for untreated and antibiotic-treated biting midges and mosquitoes to gain insight in the role of midgut bacteria in virus infection and transmission. Infection rates were determined for untreated and antibiotic-treated females of the two biting midge species *C. nubeculosus* and *C. sonorensis*. When comparing *C. nubeculosus* females fed on glucose solution with females fed on glucose solution containing antibiotics, the proportion of SBV-infected females significantly increased from 11.2 to 19.6% (*χ*^2^ test, *p =* 0.02). For *C. sonorensis*, infection rates increased from 18.2% for untreated to 34.0% for antibiotic-treated females (*χ*^2^ test, *p =* 0.14; Fig. 3a; Table 2). The observed increase for *C. sonorensis* was not significant, presumably due to the lower number of tested individuals for this species. Although the infection rate was higher in antibiotic-treated *C. nubeculosus*, the median virus titer of SBV-infected biting midges was not significantly different between untreated and antibiotic-treated *C. nubeculosus* (Mann-Whitney *U* test, *p =* 0.42) and *C. sonorensis* (Mann-Whitney *U* test, *p =* 0.89; Fig. 3b; Table 2).

**Table 2:** Infection rates, transmission efficiencies, and median (ingested) titers of untreated and antibiotic-treated biting midges orally exposed to Schmallenberg virus, and untreated and antibiotic-treated *Aedes aegypti* mosquitoes orally exposed to Zika virus or chikungunya virus. Infection rates and transmission efficiencies were determined as the percentage of insects with virus in their body or saliva, respectively, out of the total number of orally exposed insects within the respective treatment. Infection rates and transmission efficiencies are presented as percentages (number of virus positive bodies or saliva samples/total number of engorged females). Titers were determined for infected biting midge bodies, for mosquitoes infected with ZIKV, and for mosquitoes with a fully disseminated infection of CHIKV. The results represent the cumulative data from three (biting midges) or four (mosquitoes) independent biological replicates

Species	Virus	Treatment	Infection (%)	Transmission (%)	Median ingested virus titers (TCID_50_/ml)	Median titer body (TCID_50_/ml)	Median titer saliva (TCID_50_/ml)
*Culicoides nubeculosus*	SBV	Untreated	11.2 (22/196)	-	-	1 × 10^3^	-
		Antibiotic	19.6 (54/275)	-	-	1 × 10^3^	-
*Culicoides sonorensis*		Untreated	18.2 (8/44)	-	-	1 × 10^3^	-
		Antibiotic	34.0 (16/47)	-	-	1 × 10^3^	-
*Aedes aegypti*	ZIKV	Untreated	12.0 (12/100)	0 (0/100)	1 × 10^3^	1.5 × 10^4^	-
		Antibiotic	9.0 (9/100)	0 (0/100)	1 × 10^3^	1 × 10^3^	-
	CHIKV	Untreated	95.0 (95/100)	21 (21/100)	2 × 10^5^	8.7 × 10^5^	1 × 10^3^
		Antibiotic	90.0 (90/100)	17 (17/100)	1.7 × 10^5^	8.0 × 10^5^	1 × 10^3^

*SBV* Schmallenberg virus, *ZIKV* Zika virus, *CHIKV* chikungunya virus. Untreated: fed with 6% glucose solution; antibiotic: fed with 6% glucose solution with addition of penicillin and streptomycin; TCID_50_/ml: 50% tissue culture infective dose per milliliter

Infection rates and transmission efficiencies were determined for *Ae. aegypti* females exposed to infectious blood meals containing ZIKV or CHIKV. No significant differences were found in infection rates between untreated and antibiotic-treated *Ae. aegypti* females exposed to ZIKV (9.0–12.0%; *χ*^2^ test, *p =* 0.64) or CHIKV (90.0–95.0%; *χ*^2^ test, *p =* 0.28; Fig. 3c; Table 2). Moreover, no differences were found between virus titers of bodies of untreated and antibiotic-treated ZIKV-infected females (Mann-Whitney *U* test, *p =* 0.29) or CHIKV-infected females (Mann-Whitney *U* test, *p =* 0.84; Fig. 3d; Table 2).

None of the saliva samples was found positive for ZIKV by CPE; therefore, no transmission was observed for any of the ZIKV-exposed *Ae. aegypti* females. No significant differences were found in transmission efficiency between untreated and antibiotic-treated *Ae. aegypti* females exposed to CHIKV (*χ*^2^ test, *p =* 0.59; Fig. 3e; Table 2). Moreover, virus titers in saliva samples of CHIKV-infected females were all lower than 10^3^ TCID_50_/ml (Fig. 3f; Table 2).

## Discussion

The aim of this study was to investigate whether gut bacteria influence arbovirus infection and transmission in insect vectors. Our data show that feeding *C. nubeculosus* with antibiotics significantly changed their gut bacterial community composition, which was associated with increased virus susceptibility. Similar treatment had no implications for virus transmission in *C. sonorensis* or *Ae. aegypti* mosquitoes.

### Gut Bacterial Communities

Antibiotic treatment significantly changed the composition of gut bacterial communities in all three vector species. *Asaia* was identified as the most dominant bacterial genus in gut bacterial communities of the untreated groups, whereas this particular bacterium was almost non-existent in the antibiotic-treated groups. A relative reduction in *Asaia* bacteria therefore may be associated with increased infection of *C. nubeculosus* with SBV. Interestingly, similar changes in the relative abundance of *Asaia* induced by antibiotic treatment in *Ae. aegypti* did not result in any changes in susceptibility to ZIKV or CHIKV. This suggests that gut bacteria may interact in a specific manner with viruses and their vectors. At this point, we cannot provide conclusive evidence on the effect of *Asaia* on the infectivity of arboviruses in mosquitoes and biting midges. Therefore, we cannot rule out the effect of bacterial gut community density or of relatively less abundant bacteria on this tripartite interaction. This uncertainty can be illustrated by our findings on gut bacteria of *C. sonorensis*, in which *Asaia* was not the dominant species although we still found a (non-significant) trend towards increased infection in the antibiotic-treated group. Untreated *C. sonorensis* gut bacterial communities were dominated by *Pseudomonas*, Acetobacteraceae, and Azospirillaceae, whereas after treatment with antibiotics, communities were dominated by *Sphingomonas* and *Delftia*. These findings point to a potential role of gut bacteria other than *Asaia* in interference with virus infection, or possible effects of overall bacterial density. *Delftia* bacteria were found in antibiotic-treated individuals of both biting midge species, whereas they were not abundant in antibiotic-treated mosquitoes. Re-introduction of specific bacteria such as *Asaia* or *Delftia* in axenic and gnotobiotic biting midges and mosquitoes would provide important insights in species-specific roles in virus-vector interactions [5, 6, 19].

Recently, several studies have shown that bacteria in the gut of laboratory-reared mosquitoes and biting midges are different from those found in field populations [60–63]. Therefore, our findings may not directly apply to field populations of mosquitoes and biting midges. While the bacterial communities of laboratory-reared mosquitoes used for this experiment did not show any effect on virus infection or replication, midgut bacterial species found in wild populations may still have an effect. Follow-up studies should focus on identification of bacterial species from field-collected mosquitoes and biting midges, and subsequently test their vector competence.

### Virus Susceptibility

After antibiotic treatment, the susceptibility of *C. nubeculosus* to SBV increased, with almost twice as many individuals infected compared with the untreated group. For *C. sonorensis* and *Ae. aegypti*, infection rates remained equal for SBV, and ZIKV and CHIKV, respectively. Moreover, no differences in virus titers were observed between any of the untreated and antibiotic-treated groups. This suggests that the virus replicative fitness remains similar even though infectivity of SBV in the gut of *C. nubeculosus* biting midges is increased after changes in the gut bacterial communities. Absence of a salivary gland barrier for some arboviruses in *Culicoides* biting midges [64–66] suggests that higher infection rates could result in increased vector competence. We, therefore, conclude that exposure of emerging biting midges to antibiotics may cause subsequent changes in the gut bacterial communities of biting midges. This could in turn increase the risk for SBV infection of biting midges and subsequent transmission to mammalian hosts.

No effect of antibiotic treatment and consequential changes in the gut bacterial community was found on virus susceptibility or replication for *Ae. aegypti* mosquitoes in our studies. Earlier studies on susceptibility of *Ae. aegypti* for dengue virus (DENV), La Crosse virus, and CHIKV showed that specific bacteria (i.e., *Serratia odorifera* and *Chromobacterium*) could influence virus replicative fitness inside the mosquito [11–14]. For instance, the bacteria *Serratia odorifera* positively influenced DENV and CHIKV in *Ae. aegypti* mosquitoes, whereas *Chromobacterium* reduced the infection of DENV in this mosquito species. In addition, the bacteria *Serratia marcescens* facilitate DENV-2 infection by cleavage of membrane-bound mucins on the mosquito’s midgut epithelial cells [67]. Here, we identified bacteria from the same families (Enterobacteriaceae and Neisseriaceae), but did not identify bacteria classified as *Serratia* or *Chromobacterium*. The discussed studies found an effect of specific bacteria on virus infection, whereas we did not observe changes in infection after manipulation of the midgut bacterial communities in mosquitoes. This does not necessarily mean that the results of earlier studies and our study are contradictory, but that interactions are likely vector-, virus-, and bacteria species–specific. These results underscore the need to further unravel the complex interactions between midgut bacteria and the infectivity of arboviruses. This will contribute to understanding the possible implications of alterations in midgut bacteria, and how specific bacteria could be used as a novel tool for the control of arboviruses [19, 68].

Comparing vector competence of different mosquito or biting midge species, it is evident that some species are better able to transmit viruses than others [34, 69–73]. This variation in vector competence is shaped by specific interactions between virus, vector, and environmental factors [74, 75]. Our findings support the hypothesis that the gut bacterial community composition of the vector can also, at least in part, explain variation in vector competence [76]. Thus, we confirm that midgut bacteria add another level of complexity that should be considered when studying the transmission of arboviruses. Future studies on vector competence of mosquitoes or biting midges should include field-collected individuals, to assess how natural-occurring gut bacteria influence their susceptibility to virus infection.

### Possible Mechanisms

The underlying mechanism of increased susceptibility of *Culicoides nubeculosus* for SBV after antibiotic treatment remains unknown and will be an important issue for future research. Several possibilities for interaction among midgut bacteria, insect vectors, and pathogens can be considered [7, 8]. First, the presence of (sufficient) bacteria could be a key factor to reduce virus infection. This could either be through activation of the vector’s innate immune responses [26, 30, 31, 77, 78] or by directly blocking pathogen interaction with the vector midgut epithelial cells [10, 79–81]. Second, specific bacteria may facilitate arbovirus infection by digestion of membrane-bound mucins on midgut epithelial cells [67]. Third, direct competition between bacteria and viruses for resources such as lipids or vitamins could affect vector competence [2]. Finally, bacterial secretion of specific anti-pathogenic molecules, such as reactive oxygen or secondary metabolites, may kill or interfere with pathogens in the midgut [14, 27–29, 79]. Given that *Delftia* was present in both antibiotic-treated biting midge species, it would be worth investigating whether they play a role in facilitation of virus infection. Facilitation of infection was shown for *Anaplasma* bacteria in ticks, where these bacteria enhance cell apoptosis, as well as the production of proteins by the vector that reduce the formation of the peritrophic matrix and biofilms, which in turn resulted in increased infection [82, 83].

As mentioned earlier, our findings point to interactions with midgut bacteria that seem specific for each virus-vector combination. It is therefore expected that bacterial species- or population-specific interactions influence virus infection more than the mere presence of bacteria in the midgut. Bacterial interaction with the vector immune responses or secretion of anti-pathogenic molecules are likely mechanisms for the observed change in infection rates after alteration of the midgut bacteria. Several papers describe the close interaction between bacteria and the innate immune responses of mosquitoes, for example, the ability of the microbiota to modulate virus infection through stimulation of the Toll or IMD immune pathway, making this a valuable direction for further research [14, 20, 30, 31].

Although changes in infection rates may be explained by the differences in bacterial communities, an effect of the antibiotic itself on virus-vector interactions cannot be excluded. Antibiotic treatment may inhibit formation of a peritrophic matrix around the blood bolus after blood-feeding [81], thereby enhancing the possibility of virus particles to interact with the midgut epithelial cells. Furthermore, it was shown that antibiotics can induce long-lasting damaging effects on muscle structure and mitochondrial metabolism in blow flies [84]. Similar effects on midgut cells may result in a “leaky gut,” which is a well-described physiological change in insect vector midgut cells that results in increased virus infection [66, 85, 86]. However, low concentrations used in this study might not be enough to cause physiological damage to insect tissues. The way in which antibiotics play a role in changing infection rates is unclear, but it can be concluded that the uptake of antibiotics by biting midges results in higher infection rates with SBV, either through a direct effect of the antibiotic or through an indirect effect of the antibiotic on microbial communities.

### The Use of Antibiotics in the Field

Although the use of antibiotics in the livestock industry has been reduced in several European countries [87, 88], the global use of antibiotics consistently increased from the year 2000 to 2015 [89]. Of the antimicrobial compounds used in food production systems, up to 80% ends up in the environment [90, 91]. For example, antimicrobial compounds are excreted into the environment by livestock animals via urine or dung, as not all antibiotics are degraded during gut passage. This results in relatively high concentrations of antibiotics in manure [92, 93], and consequently in natural habitats of biting midges and mosquitoes. The use of antibiotics in the livestock industry may, therefore, indirectly affect susceptibility of *Culicoides* vectors for arboviruses, which may result in higher transmission risk of SBV from livestock to biting midges.

## Conclusions

Antibiotic uptake and subsequent changes in gut microbial communities resulted in an almost twofold higher infection rate of the biting midge species *C. nubeculosus* for SBV, but this was not observed with *C. sonorensis*. Use of antimicrobial compounds at livestock farms might therefore have an unexpected contradictory effect on the health of animals, by increasing the transmission of viral pathogens by biting midges. No effect of antibiotic treatment and subsequent shift in bacterial community composition on vector competence of *Ae*. *aegypti* for ZIKV or CHIKV was detected. We therefore conclude that the effect of midgut bacteria of virus infection is context-dependent and virus-vector specific. Understanding the mechanisms of how (specific) midgut bacteria influence the infectivity of arboviruses in their vectors will contribute to the search for new control strategies for vector-borne diseases.

## Electronic Supplementary Material

Additional File S1. Taxonomic composition of gut microbial community of *Aedes aegypti*, *Culicoides nubeculosus*, and *C. sonorensis* kept on either glucose solution (untreated) or glucose solution with antibiotics (antibiotic-treated). (XLSX 391 kb)

Additional File S2. Taxonomic composition of microbial community present in negative control samples. (XLSX 68 kb)

Additional File S3. Scripts used for R analyses. (R 7 kb)

## Data Availability

All data generated or analyzed during this study are included in this published article and its supplementary information files. The raw sequence data have been deposited in the NCBI BioProject repository, http://www.ncbi.nlm.nih.gov/bioproject/635089.
